# Longitudinal quantitative assessment of retinal crystalline deposits in bietti crystalline dystrophy

**DOI:** 10.1186/s12886-025-03962-8

**Published:** 2025-03-17

**Authors:** Seung Jun You, Chang Ki Yoon, Un Chul Park, Kyu Hyung Park, Eun Kyoung Lee

**Affiliations:** 1 Medical Program, Seoul National University College of Medicine, Seoul, Korea; 2 Department of Ophthalmology, Seoul National University College of Medicine, Seoul National University Hospital, #101, Daehak-ro, Jongno-gu, Seoul, 03080 Republic of Korea

**Keywords:** Bietti crystalline dystrophy, Inherited retinal disease, Longitudinal study, Quantification, Retinal crystal

## Abstract

**Background:**

This study aims to quantitatively analyze retinal crystalline deposits in patients with Bietti Crystalline Dystrophy (BCD) and examine their progression over time in a longitudinal study.

**Methods:**

We retrospectively reviewed consecutive patients diagnosed with BCD at a single center. Retinal crystalline deposits were quantified from fundus photographs using semi-automated software, which divided the macular area into a central foveal circle, inner ring, and outer ring. We then analyze changes in the area and area fraction of these deposits over a two-year period.

**Results:**

The study included 30 eyes from 16 patients. Over the two-year study period, the inner ring demonstrated the statistically significant decrease in both crystal area and crystal area fraction from baseline to one year (*P* = 0.044 for area; *P* = 0.003 for area fraction) and two years (*P* = 0.003 for area; *P* < 0.001 for area fraction). Mean crystal area fraction was 1.042 ± 0.295% in the central foveal circle, 1.056 ± 0.208% in the inner ring, and 1.001 ± 0.155% in the outer ring, with no significant differences observed between the regions (all *P* > 0.05). The genotype-phenotype correlation showed that exon7del homozygotes had significantly lower baseline crystal area and area fraction, suggesting an association with more severe disease.

**Conclusions:**

The use of semi-automated software to analyze fundus photographs provided a quantitative method for assessing retinal crystalline deposits in BCD. This longitudinal study enhanced our understanding of the disease’s natural progression.

## Background

Bietti Crystalline Dystrophy (BCD) is a rare, autosomal recessive inherited retinal disease (IRD) characterized by the deposition of numerous yellow-white crystalline materials in the retina, and occasionally in the cornea, associated with atrophy of the retinal pigment epithelium (RPE) and sclerosis of the choroidal vessels [1]. The disease typically manifests between the second and fourth decades of life and patients experience night blindness, reduced visual acuity, and visual field constriction [2].

The *CYP4V2* gene has been identified as a causative gene for BCD. It encodes a novel 525 amino acid protein, a member of the cytochrome P450 family (family 4, subfamily IV, polypeptide 2), which plays a role in fatty acid metabolism [1]. This protein is expressed in various tissues, including the human RPE, retina, cornea, and many other tissue and cells, including kidney, liver, lung, and lymphocytes [2, 3]. BCD is characterized by a dysregulation of lipid metabolism due to deficiencies of lipid binding or in fatty acid desaturation or elongation, resulting in reduced conversion of fatty acid precursors to n-3 polyunsaturated fatty acids (PUFAs) [4]. These PUFAs, which are recycled by the RPE cells, are crucial components of the photoreceptor outer segments of the retina [5]. Although crystalline materials in the posterior pole are a hallmark lesion of BCD, they gradually disappear as chorioretinal atrophy progresses [6]. The specific pathology of how mutations in *CYP4V2* lead to the formation of crystalline materials and subsequent degeneration of the RPE and photoreceptors remains unclear.

A recent study highlighted significant intereye symmetry in the area and density of retinal crystalline deposits and areas of absent-autofluorescence in both eyes of patients with BCD [7]. However, this study by Liu et al. [7] was cross-sectional and could not determine the temporal sequence of changes in the retinal crystalline deposits. Given the rarity of BCD with a global prevalence of 1 in 67,000 [2, 8, 9] and the scarcity of reports on retinal crystalline deposits and disease mechanisms, more detailed information is necessary to better understand this condition. Moreover, longitudinal studies could provide clinically significant outcome measures for gene and stem cell therapy trials. This study aims to quantitatively evaluate retinal crystalline deposits using semi-automated software on fundus photographs of patients with BCD and to explore the temporal evolution of these deposits in the macular area through a longitudinal analysis.

## Methods

### Participants

We retrospectively reviewed the medical records of patients diagnosed with clinically confirmed BCD who visited the Inherited Retinal Disease Clinic at Seoul National University Hospital between January 2005 and December 2021. The study received approval from the Institutional Review Board (IRB) of Seoul National University Hospital (IRB approval number: 2105-086-1219) and adhered to the tenets of the Declaration of Helsinki. Due to the retrospective nature of the study and the use of de-identified patient data, the IRB waived the requirement for written informed consent. The clinical diagnosis of BCD was based on a typical fundus appearance characterized by abundant small, sparkling, yellow-white crystals in the posterior pole accompanied by chorioretinal atrophy, corresponding visual field defects, and full-field electroretinography (ERG) findings. We excluded patients with optical media opacities that could significantly impair fundus image acquisition or those lacking at least two consecutive sets of fundus photographs.

### Ocular examination

All patients underwent comprehensive ophthalmic examinations at initial diagnosis, as previously described [10]. These included the measurement of best-corrected visual acuity (BCVA), slit-lamp biomicroscopy, indirect fundus examination, fundus photography, spectral-domain optical coherence tomography (SD-OCT), visual field testing, and full-field ERG. Fundus photography was performed using a Vx-10 fundus camera (Kowa OptiMed, Tokyo, Japan). SD-OCT images were captured using either a Heidelberg Spectralis (Heidelberg Engineering, Heidelberg, Germany) or a Zeiss Cirrus (Cirrus 4000; Carl Zeiss Meditec, Dublin, California, USA). The structural SD-OCT acquisition protocol included 19 horizontal raster linear B-scans, each composed of nine averaged OCT B-scans (1024 A-scans per line) at 244 μm intervals, covering an area of 20° × 20°. Full-field ERGs were conducted using gold foil recording electrodes following the International Society for Clinical Electrophysiology of Vision (ISCEV) standard protocols [11]. Visual field examinations were performed using either a Humphrey Field Analyzer (Humphrey visual field Analyzer II with Swedish Interactive Threshold Algorithm standard 24 − 2 or 30 − 2; Carl Zeiss Meditec) or a Goldmann manual perimeter (Haag–Streit, Berne, Switzerland). BCVA measurements were converted to logarithm of the minimum angle of resolution (logMAR) units for statistical analyses.

### Molecular genetic analysis

Molecular genetic tests were conducted using peripheral blood samples from patients with informed consent. Genetic testing included a next-generation sequencing (NGS)-based gene panel and whole exome sequencing (WES). The NGS-based gene panel encompassed 244 genes linked to inherited retinal diseases. WES was carried out by Macrogen in Seoul, Korea. Genomic DNA samples were enriched using the Agilent SureSelect Human All Exon Kit V6 Array (Agilent, Santa Clara, CA, USA) and sequenced using an Illumina NovaSeq 6000 system (Illumina, San Diego, CA, USA). Variant interpretations adhered to the guidelines of the American College of Medical Genetics and Genomics (ACMG) [12]. If biallelic pathogenic mutations in *CYP4V2* gene were identified, a genetic diagnosis of BCD was confirmed.

### Image processing and analysis

**Fig. 1:**
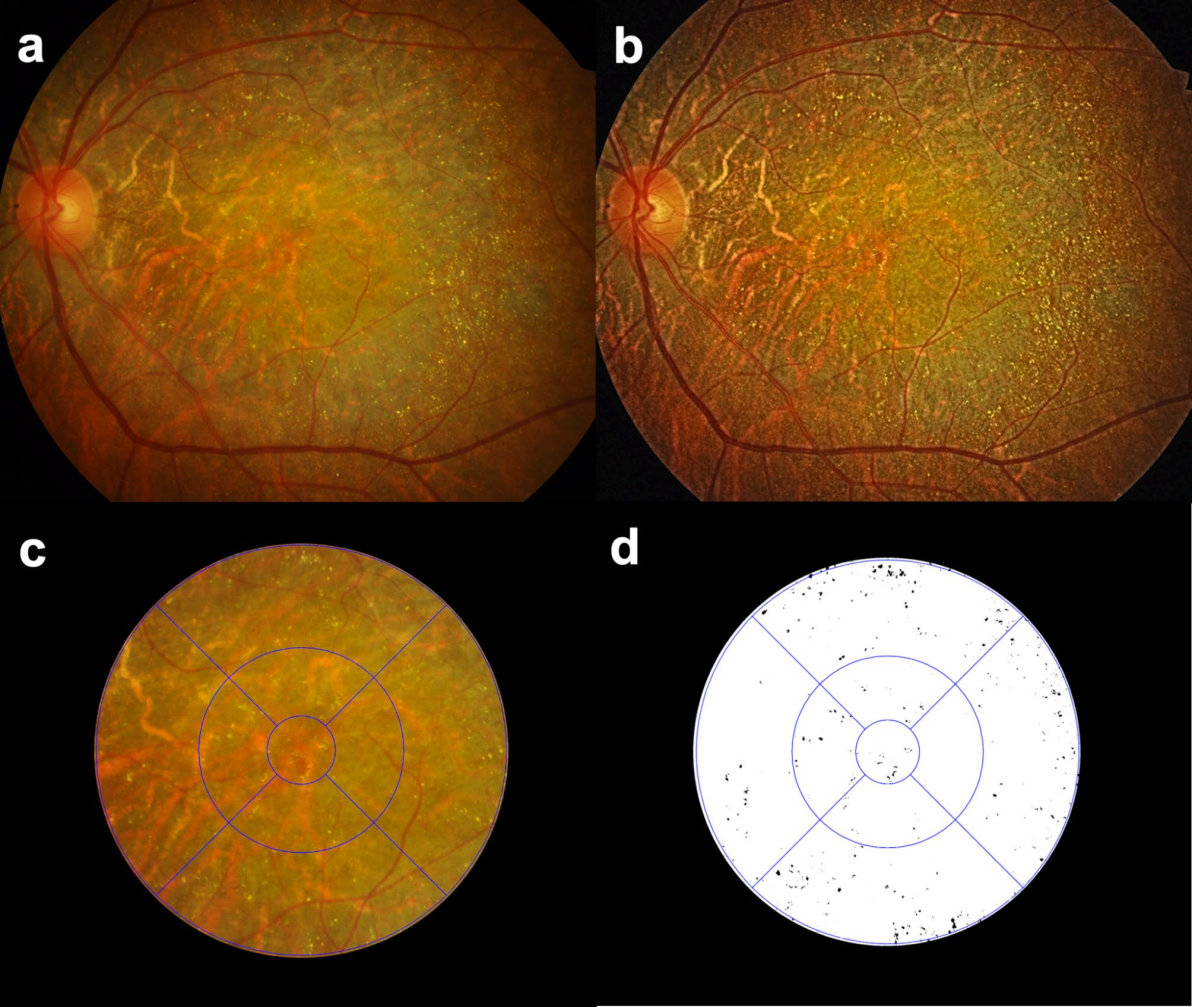
Quantitative assessment of retinal crystalline deposits in eyes with Bietti Crystalline Dystrophy. (**a**) A color fundus photograph showing greyish chorioretinal atrophy and numerous yellow-white crystalline deposits. (**b**) Image contrast enhanced using Contrast Limited Adaptive Histogram Equalization (CLAHE) to better detect retinal crystalline deposits. (**c**) An Early Treatment of Diabetic Retinopathy Study (ETDRS) grid overlaid for regional quantification of retinal crystalline deposits. (**d**) Retinal crystals extracted as black on a white background using Medilabel^®^ software

Pre-processing of fundus photography was performed to enhance the quantification of retinal crystalline deposits (Fig. 1a). Contrast Limited Adaptive Histogram Equalization (CLAHE) was utilized to isolate and detect retinal crystalline deposits in fundus images more effectively (Fig. 1b) [13]. In CLAHE, after applying a threshold filter, equalization was conducted, resulting in enhanced image contrast. The fovea was defined as a 1-mm-diameter circle, aligned with the Early Treatment of Diabetic Retinopathy Study (ETDRS) grid [14], and centered using SD-OCT guidance to achieve optimal alignment using retinal vessels as landmarks. The ETDRS grid, which includes three concentric circles with radii of 0.5 mm, 1.5 mm, and 3 mm, was overlaid for regional quantification of retinal crystalline deposits (Fig. 1c). Following, pre-processing, retinal crystalline deposits were extracted using Medilabel^®^ software (version 1.0; Ingradient Inc., Seoul, Korea). Medilabel^®^ software is a deep learning-based segmentation tool using super-pixel clustering for annotation or labeling of the target area [15]. The software generates a histogram, a graphical representation of pixel intensity distribution, enabling the identification of intensity peaks and valleys corresponding to different anatomical and pathological structures. A color fundus image was thresholded using automated histogram analysis with manual refinement to convert it into a binary image that isolates retinal crystalline deposits for quantitative assessment. The total area covered by retinal crystalline deposits was determined using an automatic threshold function, displaying retinal crystals as black on a white background (Fig. 1d). Calibration from pixels to millimeters was performed. A method of counting the number of individual crystals was initially considered, however, in some patients the retinal crystals were very close together or had a confluent clinical appearance, making it difficult to segment individual crystals. Therefore, we quantified retinal crystals by measuring the area covered by crystals and converting pixels.

Subsequently, the area of retinal crystalline deposits was quantified using the “analyze particle” function in FIJI Image J software (version 1.53; National Institutes of Health, Bethesda, MD, USA) [16]. Measurements of crystal area (mm²) were taken in different regions of the ETDRS grid: the central foveal ring (within a 1-mm diameter), the inner ring (between 1 and 3 mm annulus), and the outer ring (between 3 and 6 mm annulus). To evaluate crystal area fraction (%), the crystal area was divided by the retinal area of each corresponding region. Furthermore, crystal quantification was undertaken at baseline, 1 year, and 2 years to examine the temporal progression of retinal crystalline deposits. Two blinded graders (S.J.Y. and E.K.L.) independently evaluated all fundus images and quantification of retinal crystalline deposits. Any discrepancies were adjudicated by a senior retinal specialist, and the measurements from the two graders were averaged for statistical analysis.

### Statistical analysis

Statistical analyses were conducted using IBM SPSS version 29.0 (IBM Corp., Armonk, NY, USA). To analyze the longitudinal changes in crystal area and area fraction over time while accounting for inter-eye correlation, a Linear Mixed Model was applied with time as a fixed effect and patient identity as a random intercept. For comparing crystal characteristics between exon7del homozygotes and other genotypes, Mann-Whitney U test was used. Continuous variables are presented as mean ± standard deviation. A *P*-value < 0.05 was considered statistically significant.

## Results

### Demographics and genotype

We reviewed the medical records of 21 patients diagnosed with BCD. Of the 42 eyes examined, 12 were excluded from the analysis due to the absence of consecutive fundus photographs, leaving 30 eyes from 16 patients for inclusion. None of the patients in this cohort had a history of taking tamoxifen, canthaxanthin, chloroquine, talc, nitrofurantoin that could cause retinal crystals. The mean age of the participants was 58.31 ± 10.77 years, with the mean age at symptom onset being 46.44 ± 7.86 years. The cohort comprised five males and 11 females. Twelve patients (75.0%) presented with nyctalopia, and six (37.5%) reported a family history of IRDs. The mean initial BCVA was logMAR 1.21 ± 0.88 for the right eye and logMAR 1.28 ± 0.84 for the left eye. Regarding the central visual field (CVF), no measurable CVF with only a temporal visual field island remains in 10 eyes (33.3%), 0° < CVF ≤ 5° in 16 eyes (53.3%), and 5° < CVF ≤ 10° in 4 eyes (13.3%). The mean follow-up duration was 6.29 ± 5.40 years. Table 1 displays the demographic and clinical characteristics of the study participants.

**Table 1:** Demographics and baseline ocular characteristics of the study participants

Variable	Eyes with BCD
Age (years)	58.31 ± 10.77
Age of onset (years)	46.44 ± 7.86
Laterality, RE/LE	15/15
Sex, M/F	5/11
Nyctalopia	12 (75.0%)
Familial history	6 (37.5%)
BCVA (logMAR)	
RE	1.21 ± 0.88
LE	1.28 ± 0.84
Central visual field (°)	
No measurable CVF with only a temporal visual field island remains	10 (33.3%)
0° < CVF ≤ 5°	16 (53.3%)
5° < CVF ≤ 10°	4 (13.3%)
Follow up duration (years)	6.29 ± 5.40

BCD = Bietti Crystalline Dystrophy; RE = right eye; LE = left eye; M = male; F = female; BCVA = best-corrected visual acuity; logMAR = logarithm of the minimum angle of resolution; CVF = central visual field

Continuous variables are reported as mean ± standard deviation. All other data are numbers (percentages)

Genetic analysis was performed on 12 of the 16 participants who provided informed consent (eight using an NGS-based gene panel and four using WES). The four patients without genetic analysis were included in this study because they exhibited highly characteristic clinical findings of BCD including retinal crystals, corresponding ERG and visual field findings. Furthermore, all four patients had genetically confirmed BCD siblings, supporting their clinical diagnosis despite the absence of genetic testing. The most frequent *CYP4V2* variant identified was c.802-8_810del17insGC, with an allele frequency of 66.7%. Homozygous c.802-8_810del17insGC (p.Val268Alafs*7) mutations [exon7del] were identified in five patients, and compound heterozygous mutations of c.802-8_810del17insGC with other mutations were detected in six patients. Other pathogenic or likely pathogenic mutations included four known mutations (c.675-1G > A [p.?], c.992 A > C [p.H331P], c.810delT [p.A270fs], and c.656delT [p.F189fs]). The variant c.327 + 5G > A (p.?) was identified in two patients and classified as a variant of uncertain significance according to the ACMG guidelines, however, functional impact prediction using in-silico tools showed a higher likelihood of pathogenicity. Table 2 presents the genetic profiles of the 12 patients and the *CYP4V2* variants identified.

**Table 2:** Characterization of the disease-causing *CYP4V2* variants found

No.	Family	Allele #1		Allele #2		Zygosity
		Nucleotide (Protein)	ACMG criteria	Nucleotide (Protein)	ACMG criteria	
P1		c.802-8_810del17insGC (p.Val268Alafs*7)	P	c.802-8_810del17insGC (p.Val268Alafs*7)	P	Homo
P3		c.802-8_810del17insGC (p.Val268Alafs*7)	P	c.675-1G > A (p.?)	P	Hetero
P4	F1	c.802-8_810del17insGC (p.Val268Alafs*7)	P	c.327 + 5G > A (p.?)	VUS	Hetero
P6	F1	c.802-8_810del17insGC (p.Val268Alafs*7)	P	c.327 + 5G > A (p.?)	VUS	Hetero
P7		c.802-8_810del17insGC (p.Val268Alafs*7)	P	c.802-8_810del17insGC (p.Val268Alafs*7)	P	Homo
P9		c.802-8_810del17insGC (p.Val268Alafs*7)	P	c.992 A > C (p.H331P)	P	Hetero
P11		c.802-8_810del17insGC (p.Val268Alafs*7)	P	c.810delT (p.A270fs)	LP	Hetero
P12	F2	c.802-8_810del17insGC (p.Val268Alafs*7)	P	c.802-8_810del17insGC (p.Val268Alafs*7)	P	Homo
P13	F2	c.802-8_810del17insGC (p.Val268Alafs*7)	P	c.802-8_810del17insGC (p.Val268Alafs*7)	P	Homo
P14		c.656delT (p.F189fs)	P	c.992 A > C (p.H331P)	P	Hetero
P15		c.802-8_810del17insGC (p.Val268Alafs*7)	P	c.992 A > C (p.H331P)	P	Hetero
P16		c.802-8_810del17insGC (p.Val268Alafs*7)	P	c.802-8_810del17insGC (p.Val268Alafs*7)	P	Homo

No. = number; ACMG = American College of Medical Genetics and Genomics; P = pathogenic; LP = likely pathogenic; VUS = variant of uncertain significance; Homo = homozygous; Hetero = heterozygous

### Longitudinal quantitative analysis of retinal crystalline deposits

**Fig. 2:**
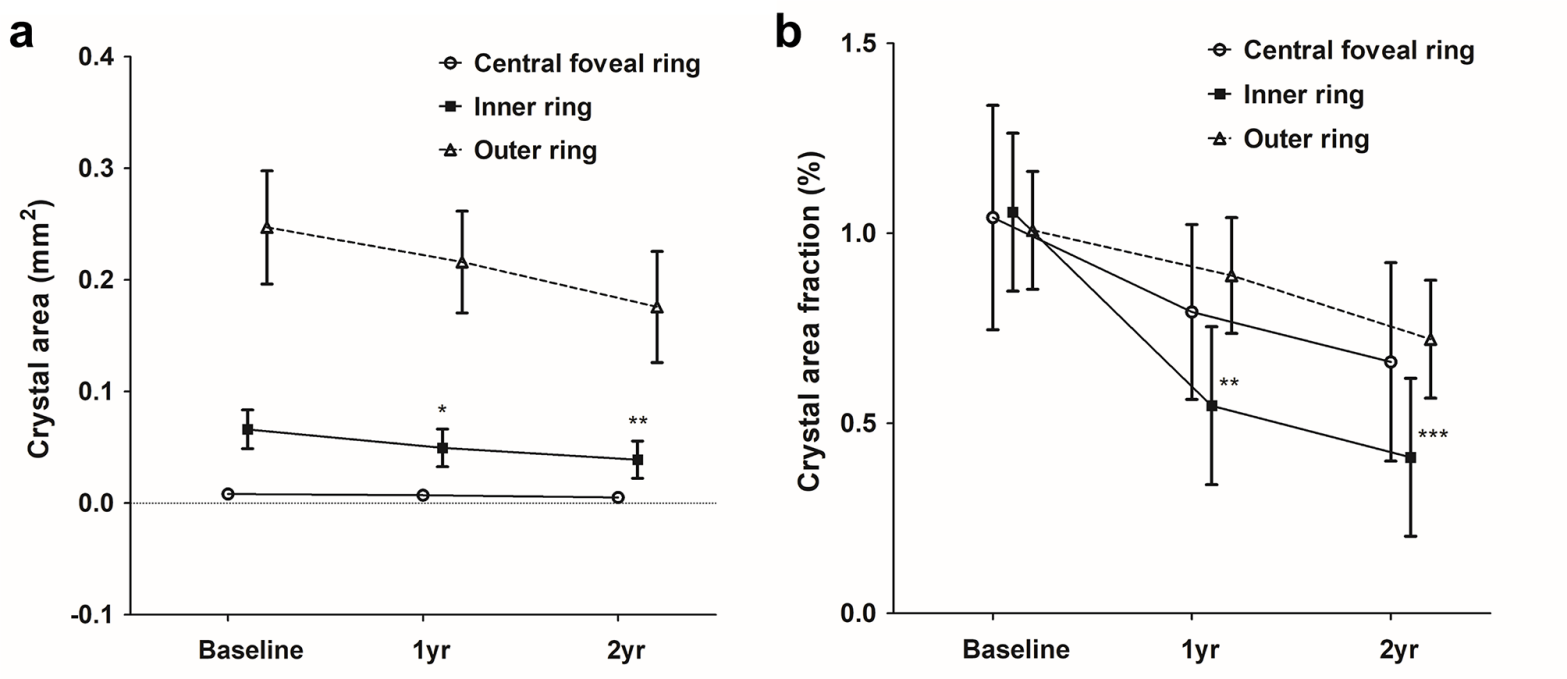
Serial changes in the average area (**a**) and area fraction (**b**) of retinal crystals throughout the follow-up period. Circles, squares, and triangles represent means, while vertical lines denote one standard error of the means from the Linear Mixed Model. P* < 0.05, P** < 0.01, P*** < 0.001

The intraclass correlation coefficient for retinal crystal area was 0.920 (95% confidence interval 0.896‒0.938, *P* < 0.001) showing high inter-observer reliability. Table 3 and Fig. 2 summarizes the serial changes in the average area of retinal crystals over time and differences in average crystal area fraction across various zones of the retina. Linear Mixed Model analysis, accounting for inter-eye correlation, demonstrated a progressive decrease in retinal crystal area across all retinal zones over the two-year follow-up period. The mean crystal area in the central foveal ring showed a modest reduction from baseline (0.008 ± 0.004 mm²) to one year (0.007 ± 0.004 mm², *P* = 0.762) and two years (0.005 ± 0.004 mm², *P* = 0.415), though these changes did not reach statistical significance. The inner ring exhibited the most pronounced and statistically significant decrease in crystal area over time (*P* = 0.006). The mean crystal area in this region significantly decreased from baseline (0.066 ± 0.017 mm²) to one year (0.050 ± 0.017 mm², *P* = 0.044) and demonstrated an even more significant reduction at two years (0.040 ± 0.017 mm², *P* = 0.003). In the outer ring, the crystal area showed a trend toward reduction from baseline (0.247 ± 0.051 mm²) to one year (0.216 ± 0.046 mm², *P* = 0.492) and two years (0.176 ± 0.050 mm², *P* = 0.151), though these changes did not achieve statistical significance.

**Table 3:** Serial changes in average retinal crystal area over time and differences in average retinal crystal area fraction across retinal zones

Crystal area (mm^2^)	Baseline	1 yr	2 yr	*P* value	Baseline vs. 1 yr	Baseline vs. 2 yr	1 year vs. 2 yr
Central foveal ring	0.008 ± 0.004	0.007 ± 0.004	0.005 ± 0.004	0.372	0.762	0.415	0.564
Inner ring	0.066 ± 0.017	0.050 ± 0.017	0.040 ± 0.017	**0.006**	**0.044**	**0.003**	0.235
Outer ring	0.247 ± 0.051	0.216 ± 0.046	0.176 ± 0.050	0.220	0.492	0.151	0.383
**Crystal area fraction (%)**	**Central foveal ring**	**Inner ring**	**Outer ring**	***P*** **value**	**Central vs. inner ring**	**Central vs. outer ring**	**Inner vs. outer ring**
Baseline	1.042 ± 0.295	1.056 ± 0.208	1.001 ± 0.155	0.981	0.967	0.914	0.851
1 yr	0.793 ± 0.230	0.547 ± 0.208	0.889 ± 0.152	0.325	0.389	0.731	0.199
2 yr	0.662 ± 0.261	0.411 ± 0.208	0.722 ± 0.155	0.296	0.447	0.832	0.240

Linear Mixed Model analysis with time as fixed effect and patient as random intercept to account for inter-eye correlation

Estimated means ± standard errors are reported from the Linear Mixed Model. Significant values with *P* < 0.05 are in bold

**Fig. 3:**
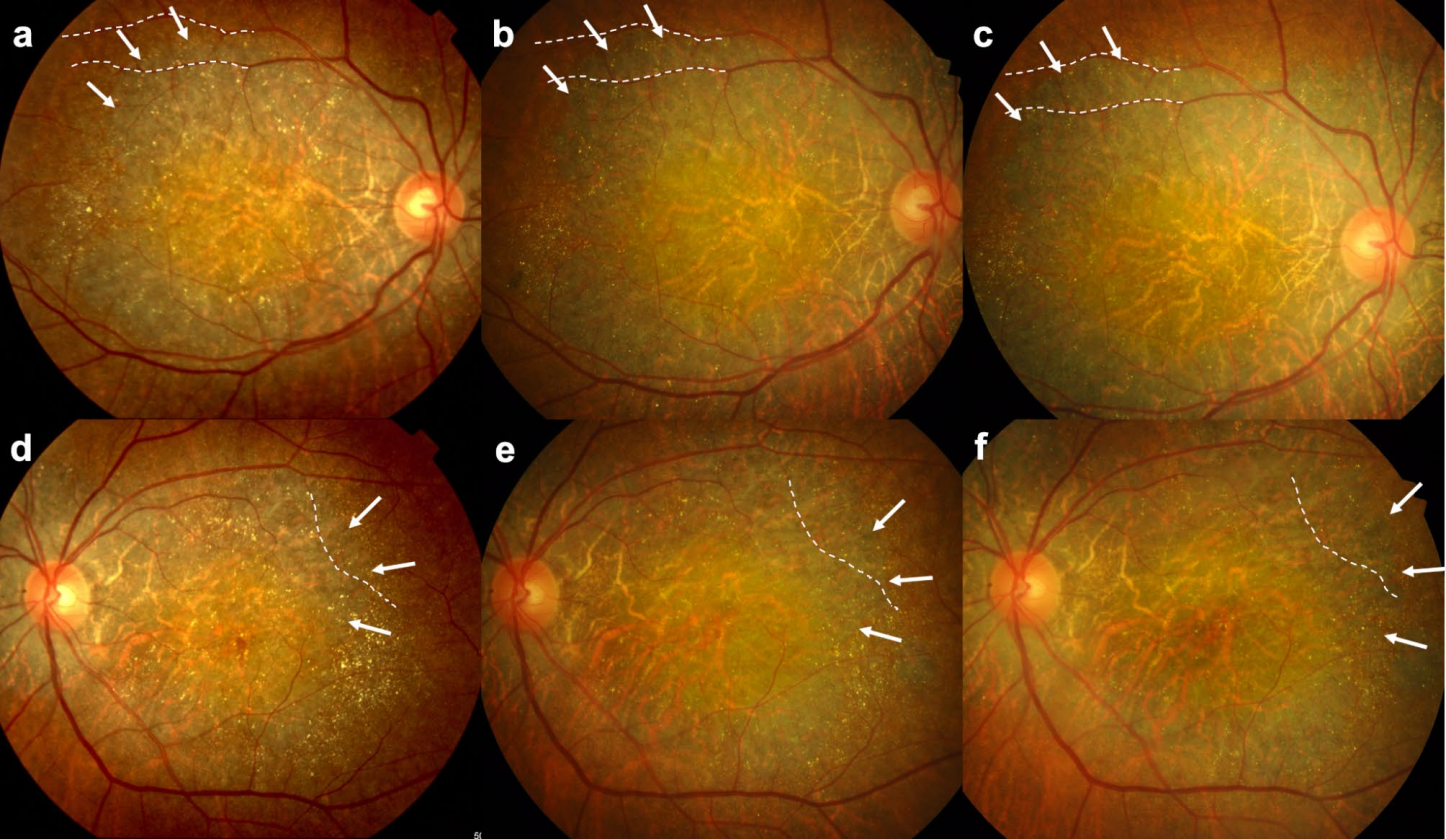
Representative case of a 69-year-old man with Bietti Crystalline Dystrophy. (**a and d**) Multiple retinal crystalline deposits observed in the macular area of both eyes at baseline. At 2.5 years (**b and e**) and 3.5 years (**c and f**), the chorioretinal atrophic region has gradually enlarged (white arrows) compared to the arrangement of retinal vessels (white dotted lines), and retinal crystalline deposits are disappearing

Similar patterns were observed for crystal area fraction. The central foveal ring demonstrated a non-significant reduction from baseline (1.042 ± 0.295%) to one year (0.793 ± 0.230%, *P* = 0.275) and two years (0.662 ± 0.261%, *P* = 0.143). The inner ring showed highly significant decrease in crystal area fraction from baseline (1.056 ± 0.208%) to one year (0.547 ± 0.208%, *P* = 0.003) and two years (0.411 ± 0.208%, *P* < 0.001). In the outer ring, a trend toward reduction was observed, with borderline significance at two years (baseline: 1.001 ± 0.155%, two years: 0.722 ± 0.155%, *P* = 0.086). Figure 3 depicts a representative case illustrating the serial changes in retinal crystalline deposits alongside the progression of chorioretinal atrophy. The pattern of retinal crystals gradually diminishes over the two-year observation period, concurrent with expanding chorioretinal atrophy. Comparison between different retinal zones at each time point showed no significant differences in crystal area fraction (all *P* > 0.05), suggesting that the crystalline deposits were relatively uniformly distributed across the macular region (Table 3).

### Genotype-Phenotype correlations and OCT image features of retinal crystalline deposits

We further analyzed the genotype-phenotype correlation by comparing the total retinal crystal burden between exon7del homozygotes and other genotypes. The total baseline crystal area was significantly lower in exon7del homozygotes (0.092 ± 0.163 mm²) compared to other genotypes (0.443 ± 0.319 mm², *P* = 0.004). Similarly, the total crystal area fraction was 1.163 ± 0.813% in exon7del homozygotes versus 4.349 ± 1.231% in other genotypes (*P* < 0.001). The rate of decrease in total crystal area over 2 years was not significantly different between groups (-58.4 ± 29.3% in homozygotes vs. -73.1 ± 12.5% in others, *P* = 0.108), suggesting that while baseline crystal burden clearly differs by genotype, the progressive dynamics of crystal reduction shows a trend that does not reach statistical significance.

**Fig. 4:**
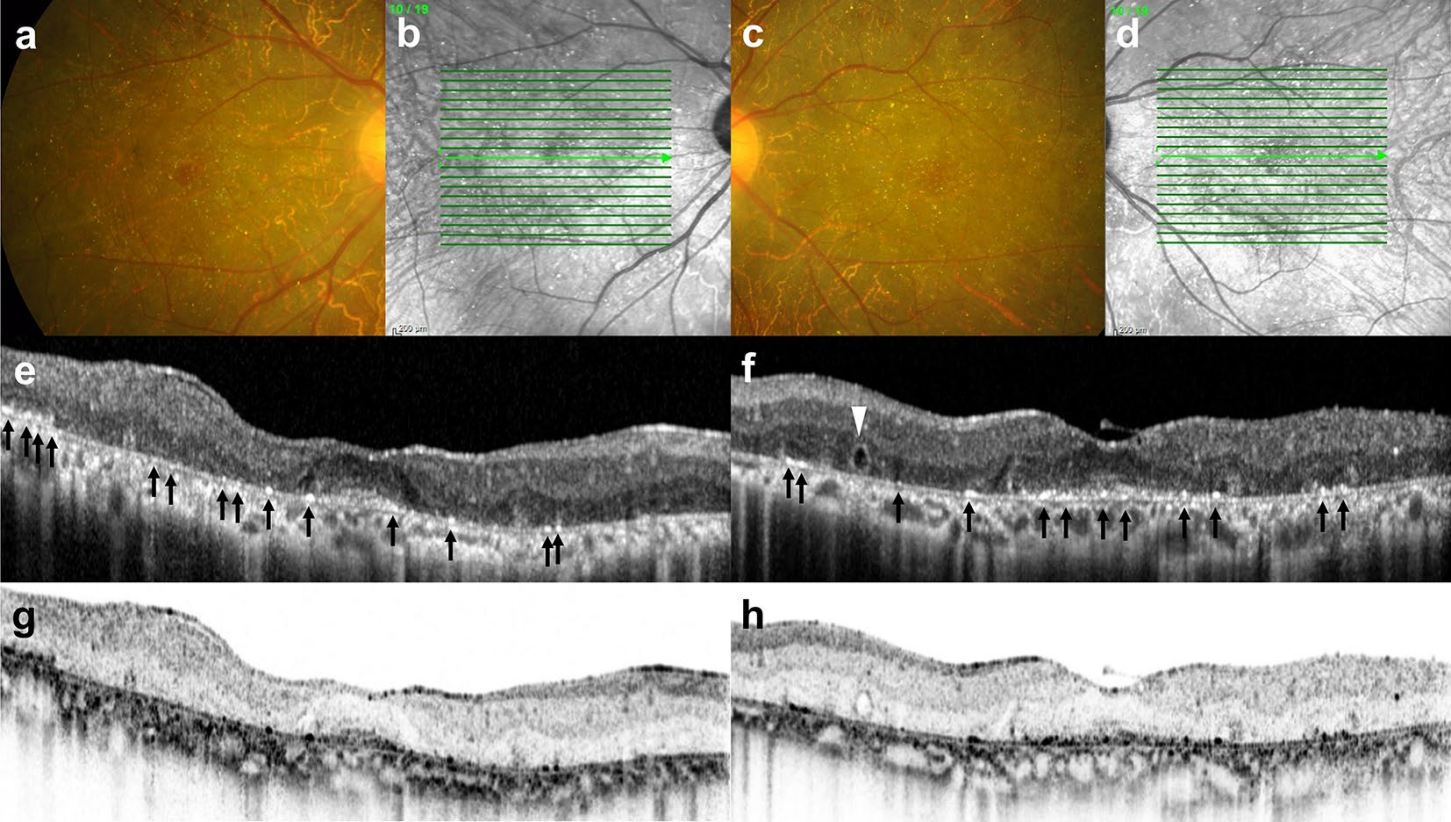
Characteristic features of retinal crystalline deposits on fundus photographs (**a and c**), near-infrared (NIR) (**b and d**), OCT image (**e and f**), and “black-on-white” image of the OCT scan (**g and h**). Note that retinal crystals were clearly seen on NIR and “black-on-white” image of the OCT scan. Green arrows in (**b** and **d**) indicate the location where the OCT scans (**e and f**) were acquired. Black arrows in (**e and f**) indicate the hyperreflective signals from the crystals that located in or on the RPE/Bruch’s membrane complex. White arrowhead in (**f**) show the outer retinal tabulation

Patients for whom OCT images were available were further analyzed to examine the characteristics and location of crystals within retinal layers. The characteristic retinal crystalline deposits and their appearance in near-infrared (NIR) and OCT images are shown in Fig. 4. The retinal crystals were clearly visualized in the NIR and the “black-on-white” image of the OCT scan. The SD-OCT scans revealed hyperreflective spots generally located in or on the RPE/Bruch’s membrane complex corresponding to the retinal crystals. We found that the retina in areas with crystals was not particularly thinner compared to adjacent areas. The crystals were rarely present in areas with loss of RPE/Bruch’s membrane and disruption of the ellipsoid and external limiting membrane layers, suggesting that crystal disappearance was associated with RPE atrophy.

## Discussion

In this study, we quantitatively analyzed retinal crystalline deposits in a patient cohort with BCD using semi-automated software. Through this longitudinal study, we observed how retinal crystals changed over time. We found that the inner ring exhibited significant decrease in both crystal area and area fraction over time. The central foveal ring and outer ring also showed trends toward reduction in crystal parameters, though these changes did not reach statistical significance. The crystal area fraction did not significantly differ between sectors of the ETDRS grid at any time point. Despite our understanding of the stages of BCD [7, 9, 17, 18], knowledge about retinal crystalline deposits remains limited. Previous studies have primarily been qualitative or cross-sectional, with little effort made to quantify crystals or examine their longitudinal changes. The clinical implications of our findings suggest that quantifying retinal crystals could make clinical assessments of BCD patients more practical and provide deeper insight into the pathogenesis and phenotype of the disease.

Crystalline retinopathy is not exclusive to BCD and can be associated with various etiological conditions, including genetic, toxic, degenerative, idiopathic, and iatrogenic factors [19]. Regarding hyperreflective crystalline deposits associated with BCD, Meyer et al. [20] reported that they appear in the retina and RPE, associated with thickening of the RPE-choriocapillaris complex in OCT images. Similarly, Halford et al. [17] observed that most crystals are located in the RPE/Bruch membrane complex, with a few crystals found elsewhere in the retina but not in the choroid. Although OCT provides detailed localization of crystals within the retinal layers, changes in the overall appearance of crystalline deposits in patients can be intuitively observed in fundus photographs. The contrast enhancement with CLAHE and the semi-automatic segmentation with Medilabel^®^ software in our study enabled us to capture crystals more effectively than with traditional color fundus photography or retinal examination.

Earlier studies on the evolution of crystals indicated that atrophy and thinning of the RPE/Bruch’s membrane complex were associated with the disappearance of the crystals. Liu et al. [7] described manual quantification methods for retinal crystals in patients with BCD and noted a strong degree of intereye symmetry. Despite being a cross-sectional study, they observed a decrease in crystal density in areas of complete RPE atrophy, suggesting a timeline where crystal appearance occurs in the early to mild stages of the disease, with crystal involution corresponding to RPE atrophy in later stages. Halford et al. [17], through serial SD-OCT imaging, also suggested that the crystals might represent a visible phenotype of metabolic dysfunction in the RPE or photoreceptor cells, with their disappearance possibly linked to cell death. Although we could not perform longitudinal quantification of RPE atrophy in this study and thus directly link it to changes in crystals, this is the first longitudinal quantitative study to show that the area fraction of crystals gradually decreased over time across all regions of the posterior pole retina, with crystal disappearance clearly evident in areas of RPE atrophy. It can be speculated that crystals are a phenotypic expression of impaired RPE function, and that regional variations in crystal number and area fraction may be useful in predicting areas of impending RPE atrophy and identifying targets for future therapeutic interventions.

Several biochemical findings have revealed systemic abnormalities of lipid metabolism in patients with BCD [4, 21], and alterations in functional *CYP4V2* are thought to cause impaired lipid processing in the RPE layer, leading to severe localized dyslipidemia and photoreceptor degeneration. Our results specifically highlight the time-dependent changes in retinal crystalline deposits, with the inner ring showing statistically significant decreases in both crystal area and area fraction over the two-year follow-up period. This regional specificity may reflect differential metabolic activity or cellular composition between retinal zones [22]. The inner ring’s pronounced crystal reduction might be related to its unique vascular supply or cellular density compared to the foveal and outer regions [23]. Interestingly, while crystals are widely considered the hallmark of BCD, our findings demonstrate their tendency to diminish over time, particularly in the inner ring, suggesting that these deposits represent a dynamic rather than static pathological feature. This progressive reduction in crystalline material raises important implications for clinical trials, as it indicates that crystal characteristics may not serve as reliable biomarkers for evaluating treatment efficacy in BCD. Future therapeutic interventions should consider alternative outcome measures that remain stable or show consistent disease progression that better reflect the underlying photoreceptor degeneration rather than the crystalline deposits themselves.

Halford et al. [17] provided the genotype and phenotype information of 20 patients with BCD and suggested that c.802-8_810del17insGC [exon7del] seemed to be related to more severe disease. In our analysis, patients with exon7del homozygotes had significantly lower baseline crystal burden compared to other genotypes, whereas there was no significant difference in the rate of crystal reduction. Our findings suggest that exon7del homozygotes seemed to be related to more severe disease, possibly as a consequence of genotype influencing initial crystal formation and accumulation, and the subsequent natural clearance process appears to follow similar biological mechanisms regardless of genetic background. These results in reduction rate is also likely attributable to the significantly lower baseline crystal burden in the exon7del homozygotes group rather than an inherent effect of the genotype on crystal clearance.

Our study had several limitations. First, the retrospective design could introduce selection bias, potentially accentuating some estimates while masking others. Second, the small sample size limited our statistical power, potentially preventing us from detecting additional significant associations and more comprehensive genotype-phenotype correlations that might exist in larger cohorts. However, given that BCD is a rare IRD and longitudinal studies on this condition are scarce, our efforts to quantify longitudinal changes in retinal crystals in patients with BCD provide a reliable foundation for future studies. Third, although we sought to minimize subjectivity by employing two different blinded graders, manual measurements of crystalline deposits remain limited. Future studies using automated crystal quantification methods are needed to confirm our results. Lastly, due to the retrospective nature of the study, we were unable to evaluate the correlation of crystal patterns with quantitative analysis of RPE atrophy on OCT and fundus autofluorescence images or visual field changes in our analyses. Further research is required to elucidate the correlation between longitudinal changes in RPE atrophy and crystalline deposits in patients with BCD, which will be essential for drawing definitive conclusions about the clinical implications of retinal crystalline deposits.

## Conclusions

In conclusion, we quantitatively analyzed the longitudinal changes in retinal crystalline deposits in patients with BCD using semi-automated software. We observed that the inner ring demonstrated statistically significant decrease in both crystal area and area fraction over time. The central foveal ring and outer ring also showed trends toward reduction in crystal parameters, though these changes did not reach statistical significance. The crystal area fraction did not significantly differ across different sectors of the posterior pole. Future studies with larger sample sizes and automated crystal quantification methods are warranted to validate and expand upon our findings.

## Data Availability

All data generated or analyzed during this study are included in this published article.

## Abbreviations

BCD: Bietti crystalline dystrophy
IRD: Inherited retinal disease
RPE: Retinal pigment epithelium
PUFA: Poly-unsaturated fatty acid
IRB: Institutional review board
ERG: Electroretinography
BCVA: Best-corrected visual acuity
SD-OCT: Spectral domain optical coherence tomography
ISCEV: International society for clinical electrophysiology of vision
logMAR: Logarithm of the minimum angle of resolution
NIR: Near-infrared
NGS: Next generation sequencing
WES: Whole exome sequencing
ACMG: American college of medical genetics and genomics
CLAHE: Contrast limited adaptive histogram equalization
ETDRS: Early treatment of diabetic retinopathy study
CVF: Central visual field
